# Effect of age and body mass index on vitamin D level in children with asthma in Riyadh

**DOI:** 10.1038/s41598-021-91108-3

**Published:** 2021-06-01

**Authors:** Iman Abdullah Bindayel

**Affiliations:** Department of Community Health Sciences, Clinical Nutrition Program, College of Applied Medical Sciences, King Saud University, Riyadh, Saudi Arabia

**Keywords:** Immunology, Medical research

## Abstract

Vitamin D deficiency prevalence in children has been rising. Low 25-hydroxyvitamin D3 (25(OH)D3) levels contribute to poor asthma control in children. This study assessed 25(OH)D3 levels in children with asthma from Riyadh with respect to anthropometrics, dietary, and lifestyle variables. Children with asthma (n, 60; 2–17 years) were assessed for serum 25-hydroxy vitamin D3 (25(OH)D3) level and body anthropometrics (weight, height, and body mass index [BMI]). Vitamin D dietary intake, sun exposure, and sociodemographic data were collected using a structured questionnaire. Thirty-one children (52%) had a 25(OH)D3 level < 50 nmol/L, 15 of whom (25%) had a level < 30 nmol/L. 25(OH)D3 level was significantly negatively correlated with age (*P* < 0.05), weight (*P* < 0.02), and height (*P* < 0.05). Children with a 25(OH)D3 level < 30 nmol/L had a significantly higher BMI than children with insufficient and sufficient vitamin D levels (*P* < 0.01). There was no significant effect of sex on 25(OH)D3 level. Higher 25(OH)D3 level was associated with a greater body area exposure to the sun. This study found that > 50% of the children with asthma had below sufficiency vitamin D levels. The vitamin D screening and supplementation of older and overweight children with asthma is recommended.

## Introduction

There has been a global rise in the prevalence of vitamin D deficiency along with an increase in asthma prevalence in children^1^. Moreover, vitamin D deficiency is associated with excessive weight in children^2^. It is speculated that children with asthma with a body mass index (BMI) > 85th percentile are at a higher risk of vitamin D insufficiency. Indeed, a study on Saudi children living in the western region found that 47% of children with uncontrolled asthma along with vitamin D deficiency were at > 95th percentile^3^. This suggests an association between vitamin D and BMI in children with asthma.

Vitamin D is required for the modulation of the immune response via the stimulation of antimicrobial protein production and its paracrine-like action on vitamin D receptors on immunocytes^4^. 25-hydroxyvitamin D3 (25(OH)D3) deficiency is linked to a higher incidence of autoimmune diseases and upper and lower respiratory tract infections^4,5^. Studies have consistently reported an inverse association between 25(OH)D3 level and worsening asthma control^3,6–9^. A serum vitamin D level < 50 nmol/L was shown to be associated with an increased inflammation and exacerbation of airways, poor lung function, and poor prognosis in patients with asthma^10^. However, a serum vitamin D level > 50 nmol/L was not associated with further improvement in asthma outcome^11^. Vitamin D supplementation is associated with an increase in the peak expiratory flow rate, a reduction in asthma severity and exacerbation, and a reduction in the risk of acute respiratory tract infection^12,13^. Therefore, vitamin D level screening and corrective treatment should be performed routinely for all children with asthma, particularly for those with predictive risk factors, such as excessive weight and older age^3,11,14,15^. There is currently no agreement on the recommended dose of vitamin D supplement that should be tailored for overweight and obese children^2^.

The prevalence of vitamin D deficiency among Saudi adults is 63%^16^, and the risk is higher among women and young adults^17^. Research examining the effect of risk factors associated with vitamin D level in Saudi children with persistent asthma is limited, particularly in the central region of Saudi Arabia. At present, two studies have been conducted on Saudi children with asthma, and both involved children living in the western region of Saudi Arabia^3,14^. However, neither of these studies collected information on dietary intake. Other studies have shown that older children with asthma and those with higher BMIs have insufficient vitamin D levels^11,14,15^.

The present study aimed to assess vitamin D status among children (age range, 2–17 years) with moderate to severe persistent asthma visiting a pulmonary clinic in Riyadh, Saudi Arabia. The relationship between vitamin D level and the related risk factors, including physical characteristics (such as age, sex, and body anthropometrics) in addition to sociodemographic, dietary, health, and lifestyle variables, was examined. Moreover, we sought to test the hypothesis that vitamin D level is influenced by predisposing risk factors in children with asthma.

## Methods

### Study participants

The aim of this observational study was to assess vitamin D deficiency in a sample of children with moderate to severe persistent asthma recruited from a pediatric pulmonary clinic in Riyadh, Saudi Arabia, over a period of 4 months (December 2017–March 2018). Ethical approval was obtained from the relevant ethics committee of the College of Applied Medical Sciences of King Saud University, Riyadh, Saudi Arabia, and all methods were performed in accordance with the relevant guidelines and regulations. A total of 61 children participants with asthma of both sexes aged 2–17 years were enrolled, and informed consent was obtained from their parents and/or legal guardians. All participating children were clinically diagnosed with moderate to severe persistent asthma by their physician as per the Global Initiative for Asthma and the Saudi Initiative for Asthma management panel guidelines^18,19^. Exclusion criteria were the presence of conditions that could potentially affect vitamin D serum level, such as kidney and liver diseases; malabsorptive syndromes in the gastrointestinal tract; and cancers. A questionnaire was administered to collect the following data: vitamin D-rich sources, fruit and vegetable intake, vitamin D supplements, sun exposure pattern, and demographic information (e.g., sex and age). The response rate was 98.7%, and only one subject refused to participate in this study owing to inconvenient time.

### Vitamin D

Total serum 25(OH)D was quantified using COBAS e-411 automated analyzer (Roche Diagnostics, Indianapolis, IN). 25-OH vitamin D deficiency was defined as a level < 30 nmol/L, vitamin D insufficiency as a level between 30 and 50 nmol/L, and vitamin D sufficiency as a level > 50 nmol/^20^.

### Anthropometrics

Weight (kg) was measured using an electronic scale and height (m) using a stadiometer, and BMI was calculated as the ratio between weight (kg) and height in meters squared. Centers for Disease Control and Prevention (CDC, 2000) growth charts for the United States were used^21^. BMI at < 5th percentile was considered underweight, 5th–85th percentile was considered normal weight, and ≥ 85th was considered overweight^22^.

### Statistical analyses

All continuous data were represented as means ± standard deviation. The sufficient, insufficient, and deficient datasets were compared using analysis of variance (ANOVA), followed by appropriate Student’s *t*-tests. A *P* value of < 0.05 was considered significant. Statistical analyses were performed using SPSS 25.0 (SPSS Inc. Chicago, IL). Binary and categorical outputs from a corresponding dietary and lifestyle questionnaire of the three 25(OH)D3 groups (sufficient, insufficient, and deficient) were analyzed using the Fisher’s exact test (< 5 cell count) or the chi-square test (> 5 cell count) and corrected using Bonferroni’s correction for multiple comparison. A *P* value of < 0.016 was considered significant. Correlations between continuous data (25(OH)D3 level, weight, height, BMI, and age) were performed using Pearson’s correlation coefficient, whereas correlations between binary and categorical data were performed using Spearman’s coefficient. The differences were considered statistically significant when the *P* value was < 0.05.

## Results

### Participant characteristics

A total of 60 children diagnosed with moderate to severe persistent asthma were included in this study. The general characteristics of the study population are presented in Table 1. Of the 60 children, 55% were males and 45% were females, and their mean age was 8.1 ± 3.4 years. More than half of the participants (58%) had normal BMI, whereas only 20% were underweight and 22% were overweight (chi-square; *P* < 0.0001).

**Table 1:** General characteristics of all participants.

Variable	Mean ± standard deviation
No	60
Sex, M/F, n (%)	33/27 (55/45)
Age, years	8.1 ± 3.4
Weight, kg	26.0 ± 11.9
Height, cm	119 ± 24.7
BMI, kg/m^2^	17.1 ± 4.3
BMI z-score	− 0.37 ± 1.81
**BMI classes**	
Underweight, n (%)	12 (20)
Normal, n (%)	35 (58)*
Overweight, n (%)	13 (22)
25(OH)D3, nmol/L	60.2 ± 39.2

Results are shown as means ± standard deviation for continuous variables.

BMI, body mass index; M, male; F, female.

*Denotes significance using chi-square *P* < 0.0001 among BMI categories.

### Sociodemographic characteristics according to 25(OH)D distribution

Participants were classified into three subgroups according to their serum 25(OH)D3 level (sufficiency, > 50 nmol/L; insufficiency, 30–50 nmol/L; and deficiency, < 30 nmol/L), and the difference among the groups was measured in relation to the variables listed in Table 2. The difference in serum 25(OH)D3 level among the groups was confirmed using ANOVA (*P* values, < 0.001, < 0.001, and < 0.001, respectively). Fifteen (25%) patients were vitamin D deficient, 16 (27%) were vitamin D insufficient, and 29 (48%) were vitamin D sufficient. There was no significant difference among the groups in the number of participants (chi-square, *P* > 0.05).

**Table 2:** Characteristics of participants according to 25(OH)D3 level (n = 60).

Variable	Sufficient	Insufficient	Deficient	*P* value
	> 50 nmol/L	30–50 nmol/L	< 30 nmol/L	
No. (%)	29 (48)	16 (27)	15 (25)	0.110
Sex (M/F), n	20/9	7/9	6/9	0.107
Age, years	7 ± 3	9 ± 4	9 ± 3	0.037
Weight, kg	23 ± 12	25 ± 10	32 ± 12*	0.043
Height, cm	114 ± 22	125 ± 19	127 ± 14	0.074
BMI, kg/m^2^	16.7 ± 4	15.5 ± 2	19.8 ± 6*†	0.011
BMI z-score	− 0.41 ± 2.0	− 1.05 ± 1.2	0.42 ± 1.79	0.075
**BMI class, n**				
Underweight	6	5	1	0.006
Normal	18	11	6	
Overweight	5	0	8‡	
25(OH)D3, nmol/L	92 ± 33	39 ± 5.9*	21 ± 4.4*†	< 0.001

Results are shown as means ± standard deviation for continuous variables.

BMI, body mass index; M, male; F, female.

*Denotes significance with respect to sufficient level using ANOVA, followed by post hoc Student’s *t*-test (significant at *P* < 0.05).

^†^Denotes significance with respect to insufficient level using ANOVA, followed by post hoc Student’s *t*-test (significant at *P* < 0.02). The difference between sex within the groups was tested using the chi-square test and corrected for multiple comparisons using Bonferroni’s correction (*P* = 0.016).

^‡^Significant difference with respect to insufficient level using the chi-square test and corrected for multiple comparisons using Bonferroni’s correction (*P* = 0.0167; *P* < 0.005).

**Figure 1:**
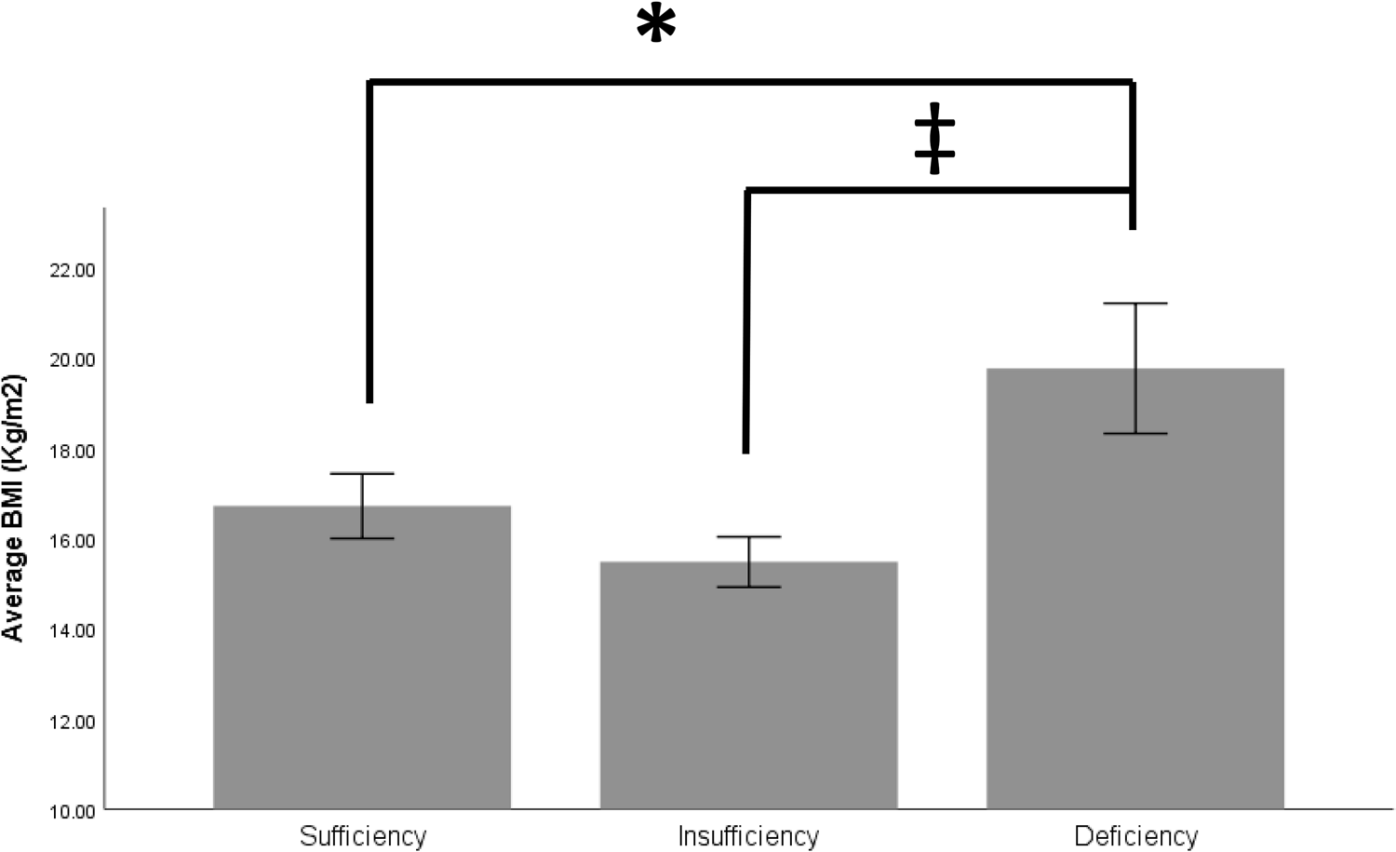
Difference in body mass index among the vitamin D subgroups. *Denotes a significant difference at *P* < 0.05; †Denotes a significant difference at *P* < 0.02.

There was no difference in vitamin D distribution between sexes (chi-square; *P* > 0.05; Table 2). Moreover, there was no significant difference between mean serum vitamin D levels between females (mean ± standard error, 55 ± 8 ng/ml) and males (64 ± 6 ng/ml) (post hoc *t*-test, *P* = 0.364). Younger participants were higher in the vitamin D sufficiency group (ANOVA, *P* < 0.05). However, the post hoc test for this group was not significant (Table 2). The deficient participants had higher weights and BMI than the sufficient participants (ANOVA, *P* < 0.05 and *P* < 0.01, respectively; post hoc Student’s *t*-test, *P* < 0.05 and *P* < 0.05, respectively) and higher BMI than insufficient participants (post hoc Student’s *t*-test, *P* < 0.01) (Fig. 1). Moreover, > 50% of the deficient group (n = 8) were at > 85th percentile, whereas none of the participants in the insufficient group and only 17% in the sufficient group were at > 85th percentile (chi-square, *P* < 0.01) (Table 2).

**Figure 2:**
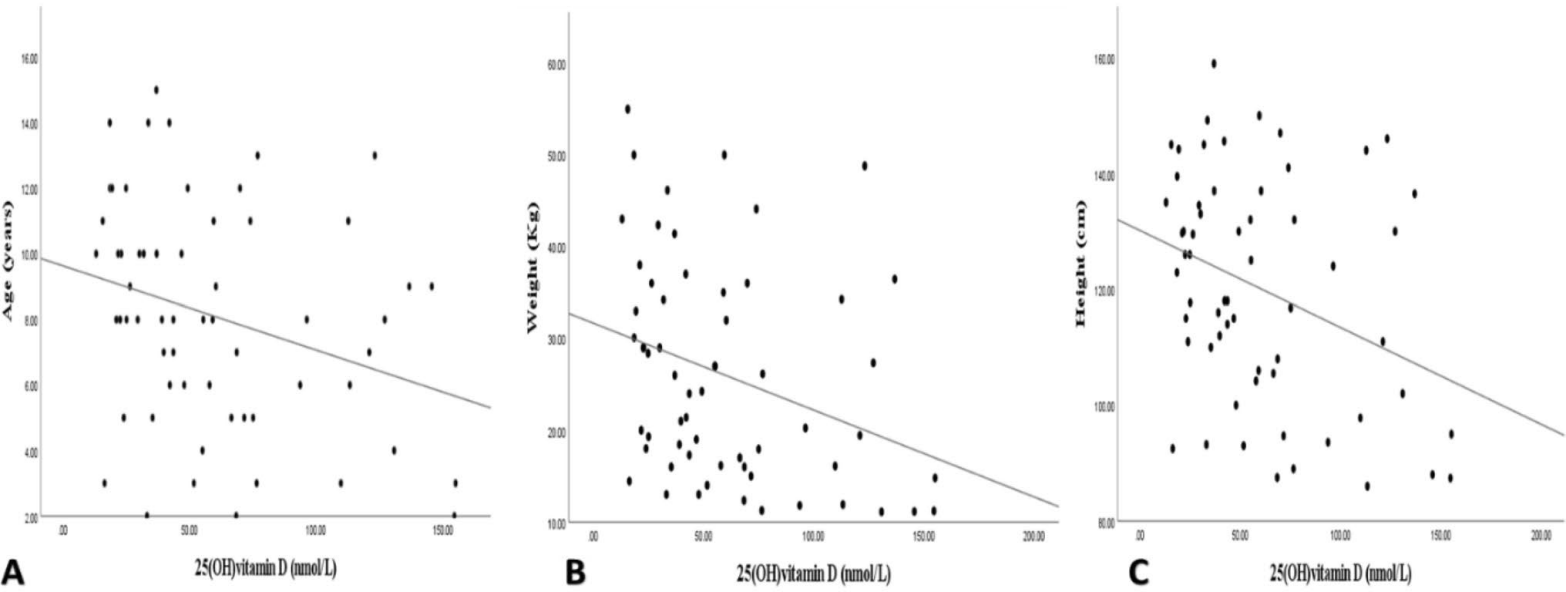
Scatter plots of serum 25(OH)vitamin D and age, weight, and height in the study population. Serum 25(OH)vitamin D level was inversely correlated with age (A, R =  − 0.293, *P* < 0.05), weight (B, R =  − 0.314, *P* < 0.02), and height (C, R =  − 0.334, *P* < 0.01).

Among the study participants, 25(OH)D3 was negatively associated with age (Pearson’s correlation: R =  − 0.293; *P* < 0.05), weight (R =  − 0.314; *P* < 0.02), and height (R =  − 0.334; *P* < 0.01) (Fig. 2). However, there was no significant association with BMI (kg/m^2^ or z-score) or sex.

### Serum 25(OH)D distribution in relation to dietary and lifestyle output

Table 3 shows the data collected from the dietary and lifestyle questionnaire for the study population according to vitamin D level distribution. Nine participants reported the use of vitamin D supplements, and the number of reporting participants was not significantly different according to vitamin D level distribution (Table 3). There was no difference among vitamin D groups in the reported presence of other illnesses (both acute and chronic), parents’ education level, knowledge about vitamin D, and knowledge regarding vitamin D sources (*P* > 0.05) (Table 3).

**Table 3:** Lifestyle and dietary patterns according to vitamin D level.

Variables	Asthmatic Children	Sufficient	Insufficient	Deficient	*P* value
No	60	29	16	15	
**Father’s educational level, n (%)**					
High School level or lower	29 (48)	16	5	8	0.051
Bachelor level or higher	31 (52)	13	11	7	
**Mother’s educational level, n (%)**					
High School level or lower	27 (45)	17	6	4	0.252
Bachelor level or higher	33 (55)	12	10	11	
Vitamin D supplements, yes, n (% yes)	9 (15)	5 (17.2)	0 (0)	4 (26.6)	0.499
Other diseases (acute and chronic), yes, n (% yes)	22 (36.7)	8 (27.5)	8 (50)	6 (40)	1.20
Difficulty walking, yes, n (% yes)	12 (20)	9 (75)	2 (17)	1 (8)	0.133
Muscle and bone pain, yes, n (% yes)	27 (45)	12 (44)	6 (22)	9 (33)	0.395
Knowledge about vitamin D, yes, n (% yes)	55 (91.7)	26 (47)	14 (26)	15 (27)	0.581
Knowledge about vitamin D sources, yes, n (% yes)	48 (80)	24 (50)	12 (25)	12 (25)	0.914
Eggs, yes, n (% yes)	51 (85)	25 (49)	15 (29)	11 (22)	0.284
Liver, yes, n (% yes)	34 (56)	16 (47)	7 (21)	11 (32)	0.245‡
Dairy products, yes, n (% yes)	59 (98.3)	29 (49)	16 (27)	14 (23)	0.348
Fish, yes, n (% yes)	36 (60)	18 (50)	9 (25)	9 (25)	0.930‡
Fruits, yes, n (% yes)	54 (90)	27 (50)	15 (28)	12 (22)	0.462
Vegetable, yes, n (% yes)	43 (72)	23 (53)	12 (28)	8 (19)	0.182
Nuts, yes, n (% yes)	37 (62)	18 (49)	10 (27)	9 (24)	0.988‡
Honey, yes, n (% yes)	38 (63)	19 (50)	10 (26)	9 (24)	0.934‡

Data are presented as n (% [percentage yes]). Categorical variables are expressed as numbers and percentages. Fisher’s exact test (two-sided) was used to compare the groups when the cell count was less than five.

^‡^Indicates the use of Pearson’s chi-square test (linear by linear correlation) to compare variables when the expected cell count was more than five (*P* < 0.05).

### Dietary intake of participants as a predictor of vitamin D deficiency

The dietary intake of the study participants is shown in Table 3. Approximately 40% participants reported no consumption of liver or fish, and the remaining children reported a liver/fish consumption of once or less per week (Table 3). In addition, eggs, fruits, and vegetables (including leafy vegetables) were not consumed daily (data not shown). Nearly all participating children reported consuming dairy on a daily basis (data not shown). The intake of nuts and honey was reported in 62% and 63% of children, respectively (Table 3). There was no difference in the reported dietary intake according to vitamin D distribution (Table 3). Moreover, there was no significant correlation between the dietary intake and serum 25(OH)D3 level (Spearman’s coefficient, *P* > 0.05). Vitamin D supplement intake was reported in only 15% of the children, but even then, nearly half of them were deficient (Table 3). However, there was no difference in reported walking difficulty or muscle or bone pain according to vitamin D distribution (Table 3). Finally, there was no significant correlation between walking difficulty or muscle or bone pain and serum 25(OH)D3 level (Spearman’s coefficient, *P* > 0.05).

### Sun exposure and its relation to vitamin D distribution

**Figure 3:**
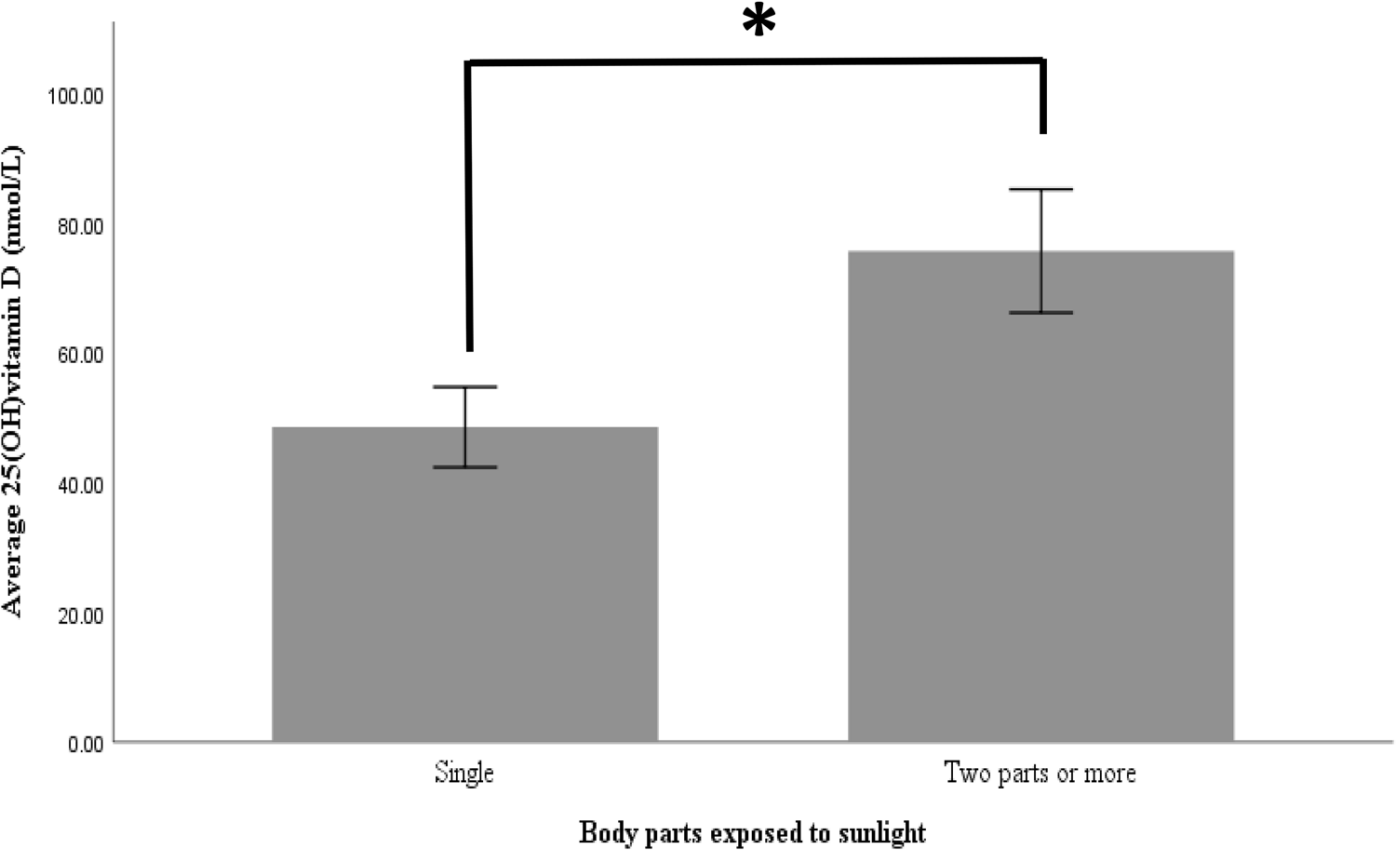
Difference in the level of vitamin D according to the number of body parts exposed to sunlight. *Denotes significant difference at *P* < 0.02.

The relationship between serum vitamin D level and sun exposure is shown in Table 4. Of the study participants, 82% of the children with asthma reported exposure to the sun. There was no difference in the ratio of participants with an inadequate duration of sun exposure (62%, 62%, and 35% in the deficient, insufficient, and sufficient groups, respectively; *P* = 0.174). However, the ratio of participants reporting multiple body part exposure was 31%, 15%, and 61% in the deficient, insufficient, and sufficient groups, respectively, indicating statistical significance (*P* = 0.02; sufficient vs. insufficient, *P* < 0.010). In addition, 25(OH)D3 level was positively associated with parts exposed to the sun (R = 0.331; *P* < 0.020) (Fig. 3).

**Table 4:** Sun exposure patterns in the children with asthma according to vitamin D level (n = 60).

Variable	N (%)	Sufficient	Insufficient	Deficient	*P* value
Sun exposure, yes, n (%)	49 (82)	23 (48)	13 (26)	13 (26)	0.912
**Time of sun exposure, yes, n (%)**					
Morning 6–10 a.m	23 (47)	11	6	6	0.838
Midday 11–3 p.m	7 (14)	2	3	2	
Evening 4–6 p.m	19 (39)	10	4	5	
**Duration of sun exposure, yes, n (%)**					
≤ 10 min	24 (49)	8 (35)	8 (62)	8 (62)	0.174‡
≥ 10 min	25 (51)	15 (60)	5(20)	5 (20)	
**Parts of sun exposure, yes, n (%)**					
Single body part	28 (57)	8 (35)	11(85)	9 (69)	0.009‡
≥ 2 parts	21 (43)	15 (65)	2 (15)*	4 (31)	

Categorical variables were expressed as numbers and percentages. Children who were never exposed to the sun were excluded (n = 11).

*Denotes significance with respect to sufficient level using Fisher’s exact test (*P* = 0.008).

^‡^Indicates the use of Pearson’s chi-square test (linear by linear correlation) to compare variables because the expected cell count was more than five (*P* < 0.05). Fisher’s exact test (two-sided) Bonferroni’s correction (*P* = 0.0167).

Serum vitamin D level was significantly higher in participants reporting the exposure of more than two body parts to sunlight versus participants reporting the exposure of a single body part (Student’s *t-*test, *P* = 0.016).

## Discussion

There is a growing health concern regarding low vitamin D status in children, particularly those with persistent pulmonary illnesses, including asthma^23,24^. This is because a low level of serum vitamin D was found to have an impact on asthma control and its severity^3,25,26^. A vitamin D level of 50 nmol/L (20 ng/ml) was adequate for improvement in asthma outcome^11^. Of note, in Saudi children with asthma living in Jeddah, 78% had vitamin D levels < 50 nmol/L (range, 10–67 nmol/L)^3^. In another study on children living in the western region of Saudi Arabia, 40% had vitamin D levels < 50 nmol/L^14^. In the present study on children with asthma in Riyadh, which was conducted based on the categorization of vitamin D levels, 52% had a level < 50 nmol/L, of whom 25% had a level < 30 nmol/L (12 ng/ml), which was the cutoff for deficiency defined in this study. This is in line with ratios reported by studies on Italian and Qatari children with asthma^11,25^. However, the ratio of sub-optimal vitamin D level in the present study is higher than that for Costa Rican and Iranian children with asthma (3.4% and 4%, respectively)^15,27^. It is speculated that the reason behind this difference in reported ratios is associated with a difference in participant characteristics, including age and BMI. The increase in the prevalence of vitamin D deficiency has been shown to be associated with a high prevalence of asthma in obese children^2^. In studies on children with asthma with normal BMI for age, lower ratios of vitamin D deficiency have been reported^15,27^. Moreover, the impact of BMI on vitamin D level is occasionally overlooked^25^.

In this study, participants with a vitamin D level < 30 nmol/L had significantly higher weights and BMIs than those with a vitamin D level > 30 nmol/L. In addition, half of the participants in the deficient group were at > 85th percentile. This is similar to the ratio reported by Aldubi et al.^3^. BMI has been shown to be associated with vitamin D level in Saudi children with asthma^14^. Children with a vitamin D level < 50 nmol/L had significantly higher BMIs than children with a level > 50 nmol/L. The impact of excess body fat on vitamin D level via the disruption of hormonal pathways hinders the synthesis of metabolically active vitamin D by the kidneys, which then impairs metabolic processes in the adipose and lean body tissues^28,29^. In addition, lifestyle factors and poor dietary habits may explain the lower vitamin D level with higher BMIs^30^. Brehm et al. reported a trend of an inverse association between BMI and vitamin D level in a population of children with asthma living in Costa Rica^15^. In addition, Bener et al. reported that high BMI is a predictor of asthma^11^. However, one study have not found an association between BMI and vitamin D^26^. This may be because of the small ratio of overweight (4% at > 85th percentile) and obese (11% at > 95th percentile) participants^26^. In addition, only a small number of children had a normal vitamin D level, whereas > 80% of the participants had a level < 50 nmol/L, with a mean vitamin D level of 14.4 ± 6 ng/ml (36 ± 15 nmol/L). By contrast, in Qatari children with asthma, the ratio for participants > 85th percentile was 19%^11^.

In the present study, vitamin D was significantly linked to the participants’ age, height, and weight, with younger, shorter, and lower weight participants more likely to have sufficient vitamin D levels. Albar et al. reported a trend for higher vitamin D level with younger age, but this did not reach statistical significance^14^. Other studies on children with asthma reported no association between vitamin D and age^3,7,26^. However, our findings are in line with those of Brehm et al. who reported that lower age and BMI were prevalent in the highest quartile of vitamin D level^15^. In addition, other studies have reported a higher BMI among patients with asthma with lower serum vitamin D levels^31,32^. It is believed that vitamin D level is lower in individuals with a larger body size because it is distributed across a larger area^30^. Sex difference with respect to the body size is well-established, but no significant difference in vitamin D distribution was observed between sexes in this study or similar studies^3,7,14,26^. In addition, the dietary intake of food rich in vitamin D was similar across groups and did not appear to affect vitamin D distribution.

Among the reasons behind the high prevalence of vitamin D deficiency in Saudi Arabia are barriers to sunlight exposure, such as traditional concealing garments and sunscreen, inactivity and limited outdoor activity, and the insufficient intake of dietary sources^7,17^. The climate in Riyadh restricts vitamin D synthesis because of the presence of dust particles and pollution, which restrict the dermal absorption of ultraviolet light, and the extremely hot temperatures during the summer, which restrict outdoor activity. Moreover, children with asthma in Riyadh are more likely to stay indoors owing to the fear of exposure to dust and pollen^33,34^. In the present study, 82% reported exposure to the sun, and the ratio of participants with an inadequate duration of sun exposure was not statistically different according to vitamin D level. However, 25(OH)D3 level was significantly associated with the number of areas exposed to the sun. The exposure of more than two body areas to sunlight was associated with a significantly higher 25(OH)D3 level than a single body area exposure. In addition, the sufficient vitamin group had the highest ratio of participants who reported the exposure of multiple body parts (61%). The exposure of 20% of the body’s surface area is sufficient to increase vitamin D level^35^.

Diet is another factor that may impact vitamin D level. The consumption of foods that are naturally rich in vitamin D (e.g., liver, fish, and eggs) reported in this study is considered inadequate. Approximately 40% reported no consumption of liver or fish, and the remaining children reported a liver/fish consumption of once or less per week. Even with almost all parents reporting adequate knowledge regarding vitamin D and its sources, their knowledge did not translate to the nutritional intake of their children. However, most children reported a daily intake of dairy that is fortified with vitamin D. We found no correlation between serum vitamin D level and dietary product intake. Moreover, dietary patterns were not different according to vitamin D distribution. Likewise, meat, fish, eggs, and dairy products did not contribute significantly to higher serum vitamin D level in a previous study^36^. In addition, children in the present study did not meet their recommended intake of fruits and vegetables because approximately half of the children who reported their dietary intake consumed fruits and vegetables three times or less per week. Moreover, 10% and 28% of the children reported no intake of fruits and vegetables, respectively. Fruits and vegetables are a good source of antioxidants in a child’s diet. Antioxidants play an important role in regulating and enhancing the function of the immune system, which reflects the importance of consuming antioxidant-rich diet in preventing autoimmune diseases, such as asthma^37^. In this study, the ratio of children consuming vegetables rich in vitamins A and C was 43% and 78%, respectively, with a frequency of three times or less per week.

This study had a few limitations. First, the lack of a control group hinders the comparison of vitamin D level with children with normal vitamin D level. Second, the effect of seasonal variation; intrinsic factors, such as skin tone; and ethnicity on vitamin D level was not considered. Third, our sample size was relatively small (n = 60). A larger sample size would be more representative of the population. Fourth, this study used the CDC growth chart, which tends to overestimate undernutrition in Saudi children. Finally, all participants had moderate to severe asthma. Thus, the effect of the degree of asthma severity on vitamin D was not measured. However, this study considered sun exposure and nutritional factors that may affect vitamin D status and asthma. In addition, appropriate statistical analyses were performed to reduce the risk of multiplicity in testing.

A protocol for routinely screening the vitamin D status in children with asthma should be implemented. At present, there is a lack of consensus in defining the vitamin D deficiency cutoff (< 50 nmol/L vs. < 30 nmol/L). Moreover, there is no agreement on a recommended vitamin D supplement dose tailored for overweight and obese children^2,7,26^. It is hoped that the results of this study will contribute to increasing the awareness for the impact of body size in influencing vitamin D level in children with asthma. In addition, future studies may benefit from the use of more accurate techniques to measure body composition, such as dual-energy x-ray absorptiometry, with respect to vitamin D. A better study design, for example, conducting a cross-regional study, is recommended.

## Conclusion

In summary, > 50% of the children with moderate to severe asthma who participated in this study had vitamin D below the sufficiency level. As expected, normal vitamin D levels were found among participants who were younger, had a lower BMI, and who reported exposing more parts of their body to the sun. In Saudi Arabia, further experimental intervention studies are needed to completely understand the effect of body size on vitamin D and to establish a supplementation dose tailored to overweight and obese children with pulmonary diseases, including asthma.

## Data Availability

The datasets used and/or analyzed during the current study are available from the corresponding author on reasonable request.
